# Case report of a familial triple: a syndrome and review of the literature

**DOI:** 10.1097/MD.0000000000020474

**Published:** 2020-05-29

**Authors:** Federica Gaiani, Pierpacifico Gismondi, Roberta Minelli, Giovanni Casadio, Nicola de’Angelis, Fabiola Fornaroli, Gian Luigi de’Angelis, Marco Manfredi

**Affiliations:** a Gastroenterology and Endoscopy Unit, University Hospital of Parma, University of Parma; b Pediatric Clinic; c Pediatric Surgery, “Pietro Barilla” Children's Hospital, University Hospital of Parma, Parma, Italy; d Department of Digestive, Hepatobiliary Surgery and Liver Transplantation, Henri Mondor University Hospital, Créteil, France; e Pediatric Unit, Maternal and Child Department, Azienda USL of Reggio Emilia, Sant’Anna Hospital, Castelnovo ne’ Monti, Italy.

**Keywords:** achalasia, Addison disease, alacrimia, Allgrove syndrome, case report, familial cases

## Abstract

**Rationale:**

Triple-A syndrome, or Allgrove syndrome (AS), is a rare autosomal recessive disorder characterized by the alacrimia, achalasia, and adrenal insufficiency triad. Alacrimia usually starts at early infancy, while achalasia and adrenal insufficiency appear later during childhood or adulthood. Some patients may also present with the so-called Double-A syndrome (i.e., alacrimia and achalasia, or alacrimia and adrenal insufficiency); adrenal insufficiency usually represents a life-threatening event due to severe hypoglycemia. Many patients may also present other associated manifestations, such as neurological disorders. We describe, here, 2 sisters of non-consanguineous parents.

**Patient concerns:**

An 8-year-old girl was admitted to the Pediatric Care Unit of Parma after an episode characterized by seizure with loss of consciousness and generalized hypertonia lasting a few minutes. Her sister, a 6-year-old girl, presented with recurrent episodes of vomiting and failure to thrive.

**Diagnoses:**

Both children were investigated by laboratory tests, esophagogastroduodenoscopy, and imaging. The first patient had the complete triad of AS (alacrimia, achalasia, adrenal insufficiency), while the second one presented only alacrimia and achalasia. Both resulted from a mutation in the achalasia, addisonianism, alacrimia syndrome gene.

**Interventions:**

Both patients were treated with oral hydrocortisone for Addison disease, and with artificial tears in the first case. After many pneumatic endoscopic dilations and therapy with nifedipine, both patients underwent surgical Heller myotomy for achalasia.

**Outcomes:**

A rapid and favorable recovery to normal diet and with improvement of growth parameters was obtained. These cases are also compared with the literature data, reported in a brief review.

**Lessons:**

AS is a rare multisystemic disorder. The longer diagnosis is delayed, the greater extent to which this syndrome may be life-threatening, mainly because of hypoglycemia due to adrenal insufficiency. In AS, the red-flag symptom of alacrimia should instigate investigation for achalasia, Addison disease, and achalasia, addisonianism, alacrimia syndrome gene mutation.

## 1 Introduction

Triple-A syndrome, or Allgrove syndrome (AS) (OMIM #231550), first described by Allgrove in 1978,^[1]^ is a rare disease characterized by adrenal insufficiency, achalasia, and alacrimia, and it can be variably associated with autonomic nervous system dysfunction and neurodegeneration. AS is an autosomal recessive disorder due to a mutation in the achalasia, addisonianism, alacrimia syndrome (AAAS) gene localized on chromosome 12q13 and codifying a protein called alacrimia, achalasia, adrenal insufficiency, neurologic disorder (commonly referred to as ALADIN), which is involved in transductional intracellular pathways.^[1,2]^

The characteristic triad of AS may manifest incompletely, but at least 2 features are necessary to make the clinical diagnosis, then being the so-called “double-A syndrome”.^[3]^ When autonomic nervous system dysfunctions are present, followed by a central and peripheral nervous system involvement (muscular atrophy, fasciculation, hypoesthesia, nasal speech, ataxia, optic atrophy, and intellectual impairment), the so-called “4A syndrome” is diagnosed.^[4–8]^ Moreover, when spinal amyotrophy appears, we can talk about “5A syndrome”.^[9]^

In AS, the onset of clinical features may not be simultaneous. Alacrimia represents the earliest and the most constant symptom, and it usually presents at birth. The symptoms of achalasia, the main complaint leading to specialist consultation, and adrenal insufficiency, due to glucocorticoid secretion deficiency, appear later during childhood or adulthood. Adrenal insufficiency is reported in 85% of patients, whereas mineralocorticoid impairment affects only about 15% of the patients. According to the overall literature data, the full triad is found in almost two-thirds of patients, 2 symptoms in one-third, and 1 symptom alone in less than 10%.^[10,11]^

## 2 Methods

We retrospectively analyzed the cases of 2 pediatric patients with AS diagnosed, treated, and followed-up in our institution since. This case presentation was conducted in accordance with the Declaration of Helsinki. The CARE guidelines were followed in accordance with the journal policies.

### 2.1 Case 1 presentation

An 8-year-old girl was admitted to the Pediatric Care Unit of Parma after an episode characterized by seizure with loss of consciousness and generalized hypertonia lasting a few minutes.

#### 2.1.1 Physical examination

Glasgow coma scale score was 10/15, and the patient did not react to verbal stimuli. Temperature and vital signs were normal. The physical examination showed xerosis cutis, skin hyperpigmentation, and no subcutaneous fat thickness; she had a normal vesicular murmur associated with crackling lung sounds. Heart rate and heart sounds were normal.

#### 2.1.2 Laboratory examinations

C-reactive protein was slightly increased (24.4 mg/L; normal range: 0–5 mg/L) and blood glucose level was very low (20 mg/dL; normal range: 60–100 mg/dL). The patient was treated with a glucosaline solution, and experienced a progressive restoration of consciousness. The immunological pattern (lymphocytes and serum immunoglobulin, islet cell auto-antibodies, enzyme glutamic acid decarboxylase, antithyroglobulin antibodies, antithyroperoxydase antibodies, anti-adrenal autoantibodies), angiotensin converting enzyme and tubercolin skin test results were all within normal limits.

#### 2.1.3 Imaging examinations

The electroencephalography, electrocardiography and brain CT-scan findings, blood cell count, liver and renal function markers, and electrolytes were normal. The brain MRI showed a normal pattern of cerebral tissue, while the optic nerve appeared presumably reduced in thickness. On the contrary, electromyography was normal.

#### 2.1.4 Past and familial history

The patient is the third of 4 children of Moroccan non-consanguineous parents. She was born via natural vaginal delivery after a full-term, uncomplicated pregnancy. The parents reported a normal achievement of psychomotor milestones. The patient received all routine childhood vaccinations, took no medications, and had no known allergies. At time of diagnosis, she had a living, apparently healthy, younger sister. Two other siblings died in Morocco due to unknown causes, at the ages of 18 months and 6 years, respectively. The father was in apparent good health, and the mother had undergone cardiosurgical valvuloplasty and pacemaker implantation to address an unspecified cardiac disease.

#### 2.1.5 Further diagnostic work-up

Because of the extremely low blood glucose level and the hyperpigmentation, the patient's endocrinological features were investigated. An extremely high blood adreno-cortico-tropic hormone (ACTH) level (2645 pg/mL; normal range: 5–49 pg/mL) and low blood cortisol level (2.0 μg/dL; normal range: 6.7–22.3 μg/dL) were found; therefore, Addison disease was diagnosed. Normal mineralocorticoid activity was confirmed by physiological aldosterone levels.

**Figure 1:**
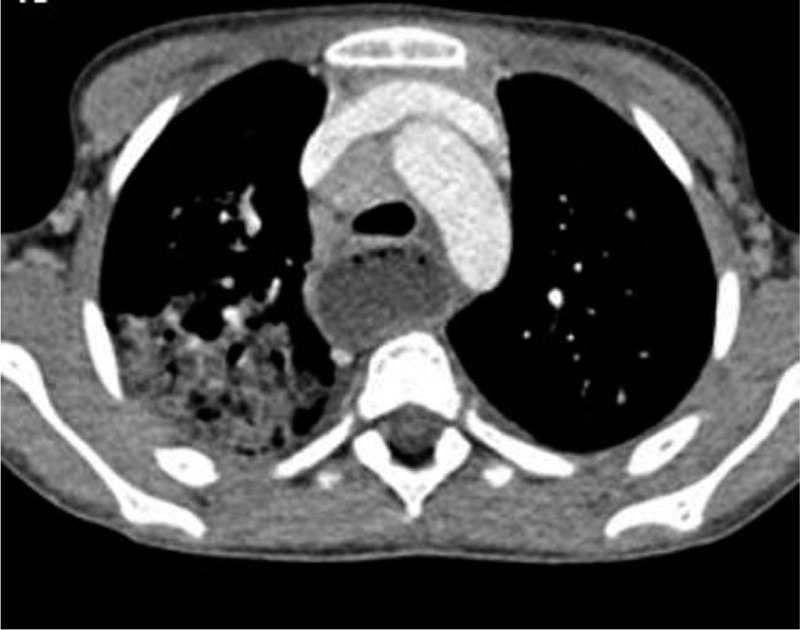
Chest computed tomography scan showing right parenchymal lobar pneumonia associated with air bronchogram, diffuse “ground glass” images, multiple mediastinal enlarged lymph nodes, and a dilated esophagus filled with solid material.

During the patient's admission, she presented a persistent cough, constant high inflammatory markers, and crackling bilateral lung sounds. A chest x-ray was performed, which showed bilateral pneumonia and enlarged mediastinal lymph nodes. The subsequent chest CT-scan showed right parenchymal lobar pneumonia associated with air bronchogram, diffuse “ground glass” images, and multiple mediastinal enlarged lymph nodes. Furthermore, it showed an entirely dilated esophagus filled with solid material (Fig. 1).

**Figure 2:**
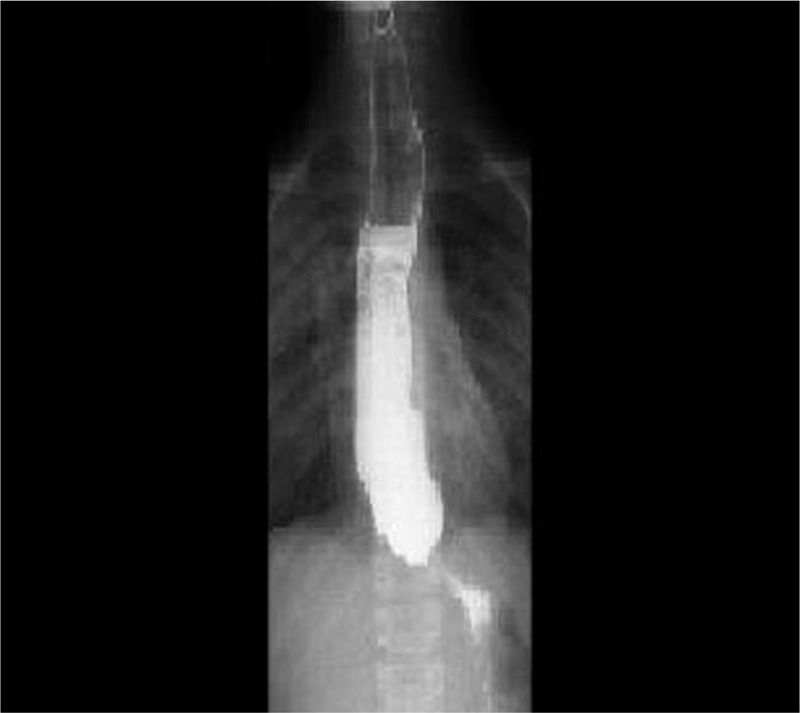
Barium swallow showing a dilation of the esophagus, narrow esophago-gastric junction with a “bird beak” appearance, aperistalsis, and a poor emptying of the barium.

After a detailed history-taking, the parents reported progressive dysphagia, failure to thrive, weight loss during the last 6 months, and crying without tears since birth. Therefore, upper gastrointestinal and bronchial endoscopies were performed, showing a dyskinetic esophagus and purulent secretion in the bronchial tree. The barium swallow showed dilation of the esophagus, narrow esophagogastric junction with a “bird beak” appearance, aperistalsis, and a poor emptying of the barium (Fig. 2). The subsequent esophageal manometry exam showed normal pressure of the upper esophageal sphincter and coordination between the pharynx and esophagus, an absent esophageal peristalsis but simultaneous uncoordinated contractions, and an augmented lower esophageal sphincter pressure, confirming esophageal achalasia.

Moreover, Schirmer test showed hypolacrimia in both eyes (< 5 mm).

### 2.2 Case 2 presentation

During the diagnostic workup, the sister of the patient presented in case 1 was investigated. She was a 6-year-old girl, presenting with recurrent episodes of vomiting and failure to thrive.

#### 2.2.1 Diagnostic work-up

The patient underwent the same diagnostic procedures as her sister (barium swallow, upper gastrointestinal endoscopy, esophageal manometry, Schirmer test), according to suspicion of the same disease. All the tests confirmed esophageal achalasia and alacrimia. The ACTH test result was within the normal range.

The coexistence of adrenal insufficiency, achalasia, and alacrimia lead to suspect AS, therefore genetic testing for AS was performed.

### 2.3 Therapeutic approach

**Figure 3:**
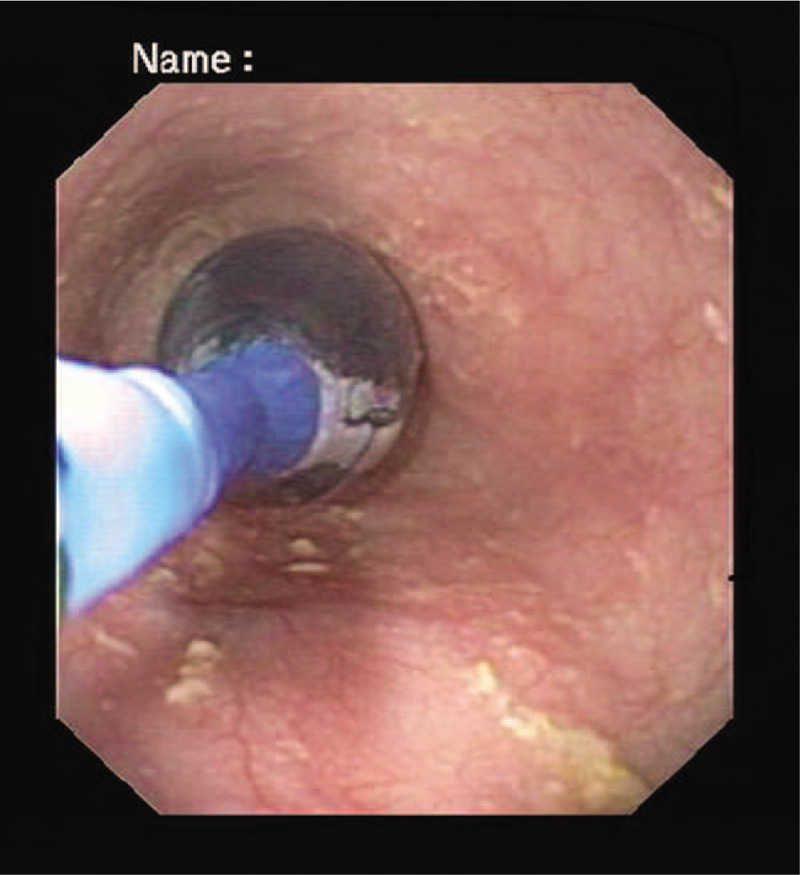
Esophageal pneumatic endoscopic dilation. The balloon was inflated under fluoroscopic control and left in the site for about 1 minute.

For the first child, a substitutive therapy with intravenous hydrocortisone was initially administered. The therapy was eventually shifted to oral administration, with no side effects. During the hospitalization, she also underwent 3 esophageal balloon dilations (Fig. 3) and began a pharmacological therapy with nifedipine (1.5 mg/kg/die t.i.d.); 17 days later, the girl started a gradual progressive oral refeeding, without dysphagia. Dysphagia and other symptoms such as drooling and weight gain were carefully monitored during the follow-up between 2 dilation sessions. The patient was discharged on therapy with oral hydrocortisone (0.75 mg/kg/die t.i.d.) and nifedipine (1.5 mg/kg/die t.i.d). A week later, the patient's conditions had improved; blood samples demonstrated a normalization of the inflammatory markers, and blood ACTH level of 1213 pg/mL. She had gained 1.5 kg of weight from the first day of admittance.

For her sister (case 2), a therapy with nifedipine was started and periodic pneumatic endoscopic dilations were performed (Fig. 3).

For both sisters, despite therapy with periodic pneumatic endoscopic dilations, frequent episodes of vomiting and the failure to thrive continued. So, a surgical laparoscopic Heller myotomy was performed without complications after 36 months from diagnosis, and 7 endoscopic dilations were performed for the first child after 25 months and 6 endoscopic dilations for her sister.

### 2.4 Final diagnosis

The final diagnosis of the 2 presented cases was AS. The syndrome was confirmed by genetic testing; both sisters were found to be homozygous for the variant c.1024 C > T (p.Arg342Term) of the AAAS gene (a nonsense mutation). For the second child, the ACTH was within normal range and the double-A syndrome was diagnosed.

### 2.5 Outcome and follow-up

Both sisters were discharged at day 7 after their surgical procedure and with a normal diet. Nifedipine was gradually withdrawn, with good compliance. The clinical examination at follow-up 6 months after surgery showed good clinical conditions, a complete tolerance of a conventional diet, and regular growth parameters.

## 3 Discussion

AS is a rare disease with still unknown prevalence and an autosomal recessive pattern of inheritance. The 3 pathognomonic clinical manifestations are alacrimia, Addison disease, and achalasia. Although some authors consider achalasia^[12]^ to be the first manifestation of the disease, alacrimia seems to be the earliest symptom,^[13]^ although it can remain undiagnosed for a long time, mainly just until an accurate history-taking is performed with parents. Other authors have stated that achalasia most probably appears later, often during adolescence or pre-adolescence.^[14]^

Alacrimia itself is a rare condition, either congenital or acquired. It can be part of familial dysautonomia, lacrimoauriculodentodigital syndrome, the anhidrotic type of ectodermal dysplasia, Sjögren syndrome, congenital disorder of deglycosylation, the alacrimia, achalasia mental retardation syndrome, and triple-A syndrome. Alacrimia is very unlikely to be an isolated finding in children; therefore, children should always be further investigated if the parents report “crying without tears”. The gold standard of diagnosis is Schirmer test, and the therapy is represented by a symptomatic approach (artificial tears and eye drops).^[14]^

Derrar et al^[25]^ described 3 African children, of 4, 5, and 7 years of age respectively, all presenting dry eye associated to achalasia with normal adrenal function. A French report^[7]^ described 14 patients affected by AS, ranging in age from 10 to 79 years, among whom the complete triad (alacrimia, achalasia, and adrenal insufficiency) was present in only 9 of the cases. Alacrimia was the earliest symptom, starting in the neonatal period. The mean age of onset of the first symptom leading to consultation was 7 years (2–13 years). However, adrenal insufficiency, achalasia and neurologic disorders were the first evidence of triple-A syndrome in 9, 3, and 2 of those patients, respectively.

Addison disease is characterized by adrenal insufficiency. In AS, the glucocorticoid function is lacking, while the mineralocorticoid function is not usually affected.

Acute adrenal insufficiency is an acute lack of glucocorticoid activity. It is usually triggered by viral infections and it is characterized by fatigue, anorexia, vomiting, abdominal pain, cyanosis, cold extremities, hypotension, increased heart rate, and weak pulse, and it can be life-threatening if misdiagnosed.^[15]^ Kurnaz et al^[16]^ evaluated 6 patients, all children of consanguineous parents and with a clinical diagnosis of AS. All of the children had both adrenal insufficiency and alacrimia, and only 4 presented achalasia. Four children presented with seizure, while 2 presented with recurrent vomiting and weakness. Low blood glucose level was present in 3 out of the 6 total children. One of these patients had a novel mutation in exon 14 of the AAAS gene.

Achalasia is a primary motor disorder of the esophagus, characterized by lower esophageal sphincter impaired relaxation and a loss of esophageal peristalsis due to an imbalance between excitatory and inhibitory neurons caused by inflammation, fibrosis, and degeneration of the inhibitory myenteric plexus. Clinical presentation varies from regurgitation of ingested food (described as vomiting immediately after meals), progressive dysphagia to solids and liquids, a deficit of growth parameters or weight loss, and repeated aspiration pneumonia.^[17]^ The diagnosis of achalasia is supported by results from barium swallow (dilation of the esophagus, narrow esophagus with a “bird beak” appearance, aperistalsis, and poor emptying of the barium) and esophageal manometry. The upper endoscopy procedure aims to assess the proximal dilatation of the esophagus and the presence of retained food material in the distal esophagus, and to exclude any other diseases (i.e., esophagitis, *Trypanosoma cruzi*, malignancy, and other secondary causes of achalasia).^[18]^

Esophageal achalasia usually requires a combined approach that includes endoscopic pneumatic dilations and a pharmacological therapy with nifedipine, with eventual surgery (Heller myotomy) in cases in which cardias stenosis persists. Actually, patients affected by achalasia usually undergo several endoscopic dilations during their lifetime, but most cases eventually require surgical intervention. Nifedipine is only a short-term therapy because it does not correct the anatomical condition, and it is associated with possible side effects (flushing and hypotension). Recently, botulinum toxin injection into the lower esophageal sphincter has been suggested, exploiting its relaxing activity on muscular fibers; however, its activity lasts only months to years and it is only a transient correction,^[19]^ making it not often considered, especially in pediatric age patients, due to risks and the limited duration.

Considering that the presentation of AS may vary widely in terms of timing, age of onset, and coexisting disorders, the final diagnosis is often difficult and delayed.^[20]^ Available literature data and case reports confirm the rarity of this syndrome and the variety of symptoms or associated diseases, mostly neurologic disorders, abnormal skin pigmentation, or edentulism, which may even precede the cardinal clinical manifestations. Moreover, although the onset in pediatric age and familiarity are usual, reports also describe adult-age onsets and unrelated cases.^[21–23]^ A review of the literature is presented in Table 1.

**Table 1:**
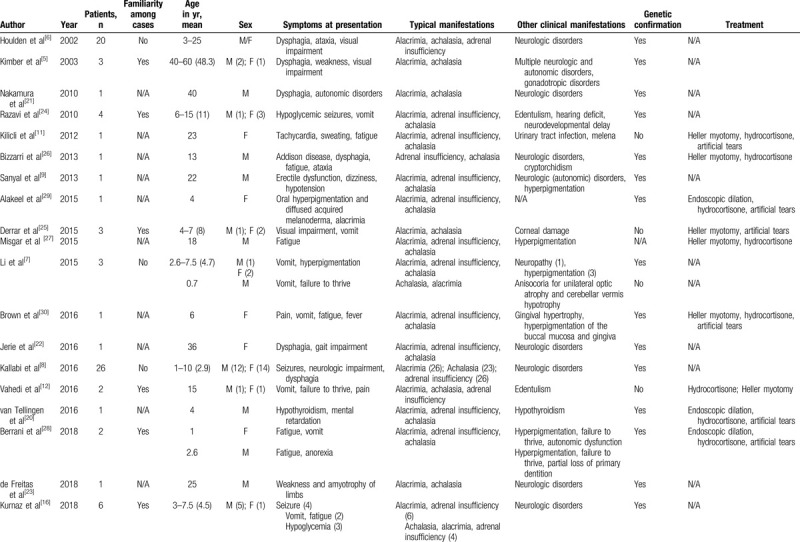
Review of the literature, case reports, and case series of Allgrove syndrome.

Several papers have reported on hypoglycemic seizures as the first clinically relevant event,^[5,14,24]^ while other patients come to the attention of clinicians due to the onset of various neurologic disorders, such as optic neuropathy, amyotrophy, gait impairment, or ataxia.^[23,25,26]^

Tibussek et al^[10]^ described 2 unrelated cases affected by AS. The first involves a 3.5-year-old girl hospitalized for repeated hypoglycemic myoclonic events. The diagnostic exams that followed revealed adrenal insufficiency and alacrimia (present since early infancy) but no achalasia. The genetic research confirmed the presence of the AAAS gene mutation. The second patient, an 8-month-old boy suffering from anisocoria, was diagnosed with unilateral optic atrophy and cerebellar vermis hypotrophy. Because of several episodes of vomiting and failure to thrive, achalasia was also diagnosed. The boy's mother reportedly mentioned the absence of tears since birth, leading to the clinical diagnosis of AS. However, the results of investigation of the common associated genetic mutations was negative.^[14]^

Edentulism^[12,24]^ and abnormal skin pigmentation^[7,27–30]^ are other rare and unusual presentations, which should however lead to further investigations for AS.

Vahedi et al^[12]^ described a young edentulous boy with the triad of AS suffering from pain associated with dysphagia, vomiting, and weight loss. After surgical Heller myotomy and supplementation of hydrocortisone, the boy's symptoms, and growth rate improved, so that he achieved a normal development. This patient had 1 sister with the same clinical characteristics.^[12]^

Brown et al^[30]^ described a 6-year-old girl who presented with diffuse intermittent abdominal pain, vomiting, fatigue, and fever. The medical history of the patient revealed a presumed diagnosis of gastroesophageal reflux at the age of 5 years. Diagnostic tests showed adrenal insufficiency with gingival hypertrophy, and hyperpigmentation of the buccal mucosa and gingiva. At 2 years of age, she stopped having overflow tears. The genetic research confirmed the mutation in the AAAS gene.^[30]^

## 4 Conclusion

AS is a rare multisystemic disorder. The longer diagnosis is delayed, the greater extent to which this syndrome may be life-threatening, mainly because of hypoglycemia due to adrenal insufficiency. Clinically, alacrimia can be considered the red-flag symptom of AS,^[15]^ although the disorder could remain undiagnosed for a long time. Moreover, other unspecific associated disorders could precede the cardinal symptoms of AS, among which some could be considered more suspicious, such as edentulism and abnormal hyperpigmentation. Overall, in all patients presenting alacrimia, mutations of the AAAS gene should be always verified to avoid diagnostic delay of AS. On the contrary, achalasia, and adrenal insufficiency usually begin later, during childhood or adolescence, although often before the neurological manifestations.^[3,31]^ Each of these pathognomonic signs and symptoms should always be considered in association and carefully investigated, as the earlier and more precise is the diagnosis, the better is the clinical outcome.

## Acknowledgments

We gratefully acknowledge the patients and their family for collaboration.

## Author contributions

Gaiani F, Gismondi P, Manfredi M and de’Angelis GL conceived of the study; Gaiani F, Manfredi M, Casadio G and Fornaroli F wrote the manuscript; Minelli R, de’Angelis N and de’Angelis GL revised the manuscript for important intellectual content. All authors approved the final version.
